# Antibacterial Activity and Mechanism of *Litsea cubeba* Essential Oil Against *Salmonella typhimurium*

**DOI:** 10.3390/plants14091343

**Published:** 2025-04-29

**Authors:** Cuncai Wang, Xiying Chen, Mingjie Liu, Xiaoquan Tang, Youzhi Li, Yuming Zhan, Zhihui Hao

**Affiliations:** 1 National Key Laboratory of Veterinary Public Health Safety, College of Veterinary Medicine, China Agricultural University, Beijing 100193, China; b20213050439@cau.edu.cn (C.W.); b20233050486@cau.edu.cn (X.C.); 13201273636@163.com (M.L.); 15970798816@163.com (X.T.); 2 Key Biology Laboratory of Chinese Veterinary Medicine, Ministry of Agriculture and Rural Affairs, Beijing 100193, China; 3 Innovation Centre of Chinese Veterinary Medicine, College of Veterinary Medicine, China Agricultural University, Beijing 100193, China; 4 Shandong Provincial Key Laboratory of Quality and Safety Monitoring and Risk Assessment for Animal Product, Shandong Center for Quality Control of Feed and Veterinary Drug, Jinan 250100, China; sdsy8015@163.com (Y.L.); 13589018038@163.com (Y.Z.)

**Keywords:** *Litsea cubeba* essential oil, *Salmonella typhimurium*, antibacterial, anti-biofilm

## Abstract

*Litsea cubeba* essential oil (LCEO) has been reported as an antibacterial agent, but its effects against *Salmonella typhimurium* (S.Tm) and the underlying mechanisms remain unclear. The antibacterial efficacy of LCEO was assessed utilizing both microdilution and growth curve methodologies, and its chemical composition was thoroughly analyzed. Morphological alterations in the cells were observed through scanning electron microscopy (SEM), while cellular permeability was gauged based on the variations in nucleic acid and protein contents. The impact of LCEO on ATPase activity and its anti-biofilm formation activity was assessed using colorimetric methods. The results indicated that the MIC and MBC of LCEO against S.Tm were 0.4 mg/mL and 0.8 mg/mL, respectively. SEM and PI staining revealed disrupted bacterial cell integrity. Compared to those in the control group, treatment with LCEO significantly elevated the levels of extracellular nucleic acids and proteins (*p* < 0.05). Furthermore, at the MIC, LCEO led to a 77.9% reduction in AKP content, and decreased intracellular Na^+^K^+^-ATPase and Ca^2+^Mg^2+^-ATPase activities by 79.9% and 54.6%, respectively. Additionally, LCEO markedly inhibited biofilm formation, enhanced surface hydrophobicity, and diminished the swimming motility of S.Tm. Overall, LCEO exhibited promising antibacterial properties, indicating its potential as an effective inhibitor against S.Tm.

## 1. Introduction

Foodborne pathogens can accelerate feed deterioration, lead to economic losses, and contribute to foodborne diseases that pose significant risks to livestock health [1,2,3]. Common foodborne pathogens include *Salmonella typhimurium* (S.Tm), *Escherichia coli*, *Listeria monocytogenes*, and *Bacillus cereus*, among others [4,5,6,7]. Notably, S.Tm is one of the most frequently isolated bacterial types globally, with contaminated feed often serving as the source of infection for both humans and animals. Survey reports indicate that S.Tm contamination is prevalent in most regions worldwide, posing substantial threats to public health and resulting in significant economic losses [6,8]. Although antibiotics are widely used as feed additives to mitigate and control feed poisoning and deterioration associated with microbial growth, their excessive use has led to strong drug resistance among many foodborne pathogens [9], posing serious threats to human and animal health. In light of the ban on antibiotics as feed additives and the current consumer demand for products free from chemical antimicrobials, natural antimicrobial substances emerge as the most promising alternative.

Essential oils, commonly referred to as volatile oils, are a type of oily liquid composed of compounds such as terpenes, aldehydes, and esters, and belong to plant-derived secondary metabolites. Current research indicates that many essential oils exhibit certain inhibitory effects on fungi and bacteria, making them suitable as natural preservatives and freshness extenders to prolong the shelf life of food products [10,11]. Previous studies have demonstrated that cinnamon essential oil can disrupt bacterial cell membranes and alter the lipid profile of these membranes, effectively killing bacteria by inhibiting their movement and biofilm formation [12]. Song et al. [13] found that citrus essential oil, when applied to *Staphylococcus aureus*, can enhance cell membrane permeability and inhibit protein production, thus exhibiting antibacterial activity. In addition, the essential oils of lemon, grapefruit, and orange have effects on some bacteria (*Lactobacillus campylobacter*, *Lactobacillus sakai*, *Staphylococcus sarcosus*, and *Staphylococcus xylosus*) and food-borne bacteria (*Escherichia coli*, *Staphylococcus aureus*, and *Listeria monocytogenes*) in the food industry [14]. Plant essential oils are considered a natural and safe alternative to synthetic preservatives and antimicrobials due to their broad-spectrum antibacterial action compared to chemical antimicrobials, and they have garnered significant attention in the food and feed additive industries.

*Litsea cubeba* essential oil (LCEO) is primarily extracted from the fruit of *Litsea cubeba*, a plant belonging to the Lauraceae family. It is characterized by a lemon flavor and pungent taste, and exhibits a wide range of pharmacological effects [11]. LCEO has been shown to protect colon tissue by maintaining epithelial integrity and reducing levels of TNF-α, IL-6, and IL-1β in the liver and intestines, thereby exerting anti-inflammatory effects [15]. Chen et al. [16] demonstrated that supplementing LCEO in feed improved growth performance and physiological and biochemical indicators in pigs, while also enhancing the body’s antioxidant capacity and nutrient digestion and absorption efficiency. Furthermore, LCEO exhibits potent antibacterial activity. Wang et al. [17] evaluated the antibacterial activity of LCEO against fungi, revealing that treatment with LCEO severely disrupted the morphology and ultrastructure of fungal hyphae, cell membranes, and organelles. Antibacterial activities have also been reported against bacterial pathogens, including methicillin-resistant *Staphylococcus aureus*, *Vibrio parahaemolyticus*, *Listeria monocytogenes*, and *Streptococcus garvieae* [17,18,19]. However, research on the antibacterial activity and mechanism of action of LCEO against S.Tm remains limited.

Previous studies have confirmed the antibacterial activity of LCEO; however, the specific effects on bacterial strains remain unclear, and there has been limited exploration of the active substances in LCEO that contribute to its antibacterial properties. Therefore, this study utilized LCEO as a natural antibacterial agent, employing S.Tm as the experimental strain. The antibacterial activity of LCEO against S.Tm was assessed by measuring the minimum inhibitory concentration (MIC) and minimum bactericidal concentration (MBC). Furthermore, the study investigated the mechanism of action, focusing on alterations in cell membrane permeability, intracellular nucleic acid concentration, and protein content, with the aim of providing insights for the development of natural antibacterial alternatives to prevent S.Tm infection.

## 2. Results

### 2.1. Chemical Composition Analysis of LCEO

The chemical composition of LCEO is presented in Table 1. A total of 21 components, including monoterpene aldehydes, alcohols, alkenes, and esters, were identified through GC–MS analysis, collectively accounting for 99.48% of the total components of LCEO. Among these, neral (29.76%) and geranial (35.62%) emerged as the primary compounds, together constituting 65.38% of the total content. Furthermore, 10 compounds with contents exceeding 1.00% were identified, representing 29.32% of the total LCEO content. These 10 components include α-terpineol (6.25%), ylangene (4.38%), geraniol (3.43%), α-pinene (3.18%), α-bisabolene (2.85%), α-phellandrene (2.76%), citronellal (2.11%), nerol (1.79%), 6-methyl-5-hepten-2-one (1.56%), and α-terpinene (1.01%).

The formula for calculating the retention index (RI) was as follows,

$$RI=100n+100\times \left(\frac{t_{R}-t_{n}}{t_{n+1}-t_{n}}\right)$$

where t*_R_* is the retention time of the target compound, t*_n_* is the retention time of the n-alkane with carbon number n, t*_n+1_* is the retention time of the *n*-alkane with carbon number *n* + 1, and n is the retention time of the target compound that falls between that of *n*-alkanes with carbon numbers n and *n* + 1.

### 2.2. Antibacterial Activity of LCEO

#### 2.2.1. Inhibition Zone Analysis

The DIZ, MIC, and MBC values of LCEO and *berberine hydrochloride* for different bacteria are shown in Table 2. LCEO exhibited significant inhibitory effects against *Escherichia coli*, S.Tm, *Listeria monocytogenes*, and *Bacillus cereus*, with DIZ measuring 22.1 ± 0.5 mm, 25.5 ± 0.7 mm, 20.2 ± 1.2 mm, and 18.9 ± 0.3 mm, respectively. The results indicated that LCEO exhibited antibacterial activity against all four foodborne bacteria (DIZ > 18 mm). Notably, LCEO demonstrated a more pronounced antibacterial effect against Gram-negative bacteria in comparison to Gram-positive bacteria. The MIC and MBC values of four bacteria were determined using diluted LCEO. As shown in Table 2, the MBC values for all bacteria were below 2.0 mg/mL. The MIC values for *Escherichia coli*, *Salmonella enterica*, *Listeria monocytogenes*, and *Bacillus cereus* were 0.8, 0.4, 0.8, and 0.8 mg/mL, respectively. Qualitative experiments showed that LCEO almost completely inhibited the growth of S.Tm, and quantitative results showed that it had the lowest MIC and MBC values. Therefore, S.Tm was selected as the experimental object for subsequent analysis.

#### 2.2.2. Time-Killing Curves

**Figure 1:**
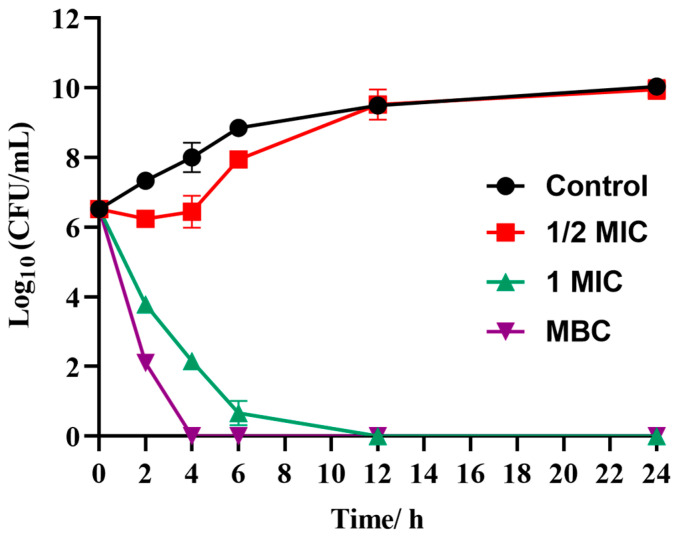
Inhibition curve of LCEO (1/2 MIC, MIC, and MBC) against S.Tm time–sterilization curve.

Based on the MIC and MBC test results (Table 2), a time-killing curve was used to describe the feasibility of LCEO in killing S.Tm. As shown in Figure 1, three LCEO concentrations (1/2 MIC, MIC, and MBC) were used to determine their ability to kill S.Tm, within 24 h. S.Tm treated without LCEO was a blank control. The results indicated that at 1/2 MIC, the antibacterial effect of LCEO was weak and could not effectively kill S.Tm. At the MIC, the sterilization rate of LCEO reached 98.8% after 2 h and 99.98% after 12 h. At the MBC, LCEO showed a significant increase in bactericidal activity time, reaching 99.96% at 2 h and 99.99% at 4 h. Therefore, the MIC and MBC of LCEO showed a strong bactericidal effect on S.Tm, while higher concentrations increased the measurement range and had an impact.

### 2.3. Effects of Environmental Factors on the Bacteriostatic Stability of LCEO

**Figure 2:**
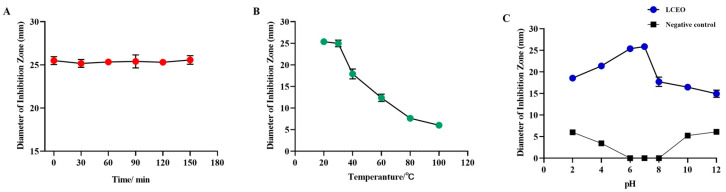
Influence of environmental factors on the antimicrobial stability of LCEO. (**A**) UV exposure. (**B**) Temperature. (**C**) pH.

As depicted in Figure 2A, LCEO showed no significant influence on the antibacterial effect of S.Tm across various UV exposure durations, signifying that the antibacterial components of LCEO remained unaffected by UV radiation, exhibiting good UV stability. As illustrated in Figure 2B, the antibacterial activity of LCEO diminished with rising heating temperatures, leading to almost complete loss of antibacterial activity against S.Tm at 100 °C. Compared to high-temperature environments, LCEO exhibited more stable antibacterial effects in low-temperature environments. Figure 2C illustrates the effect of pH on the antibacterial activity of LCEO. The negative control experiments revealed that inhibition zones began to appear when pH was less than 6 or greater than 8, reaching their maximum at pH 2 and pH 12, respectively. In environments with pH levels ranging from 6 to 8, there was no significant impact on the growth of S.Tm, indicating that extreme pH conditions affected its growth. The images of the LCEO treatment group demonstrated that LCEO exhibited substantial antibacterial activity against S.Tm, with its DIZ values significantly exceeding those of the negative control group. Furthermore, pH also influenced the antibacterial activity of LCEO. Figure 2C indicates that in environments with pH levels of 6 to 7, the antibacterial activity of LCEO against S.Tm was significantly greater than in environments with pH levels of 2 to 6 and 7 to 12. Additionally, the trend of DIZ values in the graph suggests that the alkaline environment had a more pronounced impact on the antibacterial activity of LCEO compared to the acidic environment.

### 2.4. Effect of LCEO on Morphology and Membrane Permeability of S.Tm

**Figure 3:**
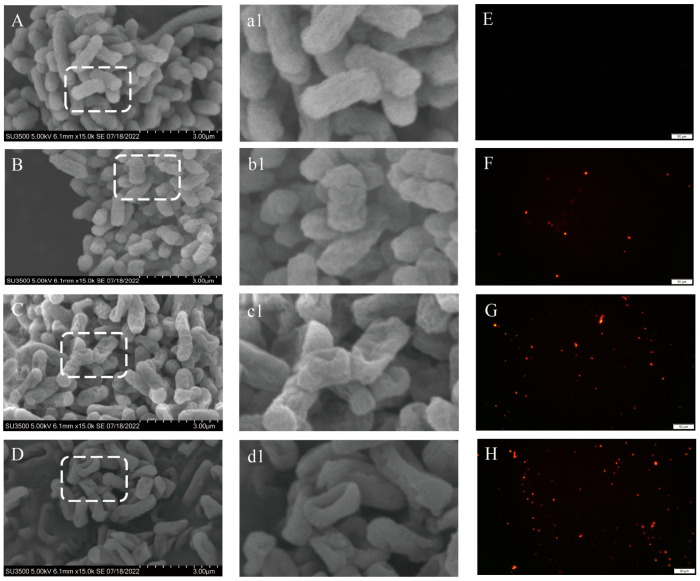
SEM and fluorescence microscopy images of S.Tm treated with different concentrations of LCEO. (**A**–**D**) SEM images of bacteria treated with LCEO at 0, 1/2MI, CMIC, and MBC, respectively. (**a1**–**d1**) Magnified part of rectangle in (**A**–**D)**, respectively. (**E**–**H**) Fluorescence microscopy images of bacteria treated with LCEO at 0, 1/2MI, CMIC, and MBC, respectively.

SEM images illustrate the effects of various concentrations of LCEO (1/2 MIC, MIC, and MBC) treatment on the morphology of S.Tm (Figure 3A–D). There was a significant difference in cell morphology between the control group and the experimental group. The untreated cells (Figure 3A(a1)) exhibited normal rod shapes with smooth and plump cell morphology. However, after treatment with 1/2 MIC, the cell surface became wrinkled, and slight adhesion was observed between the cells (Figure 3B(b1)). When the cells were treated with 1 MIC LCEO (Figure 3C(c1)), the surface of S.Tm cells became rough, the cells start to collapse, there was intercellular aggregation and adhesion, and cell boundaries became blurred. When LCEO was further increased to the MBC (Figure 3D(d1)), severe physical damage and cell lysis were observed, and cell adhesion further intensified. This suggested an increase in cell membrane permeability, leading to a significant efflux of intracellular substances. The results of PI staining further validated the aforementioned conclusion (Figure 3E–H). Compared with those in the control group (Figure 3E), bacteria exposed to 1/2 MIC (Figure 3F) exhibited partial red fluorescence, and a significant increase in red fluorescence was observed with LCEO treatment at the MIC (Figure 3G). Bacteria co-cultured with LCEO exhibited more red fluorescence, especially at MBCs (Figure 3H), indicating that LCEO disrupted the bacterial cell membrane, allowing PI to penetrate and stain the membrane.

### 2.5. Antibacterial Mechanism of LCEO

#### 2.5.1. The Influence of LCEO on the Cell Membrane Potential of S.Tm

**Figure 4:**
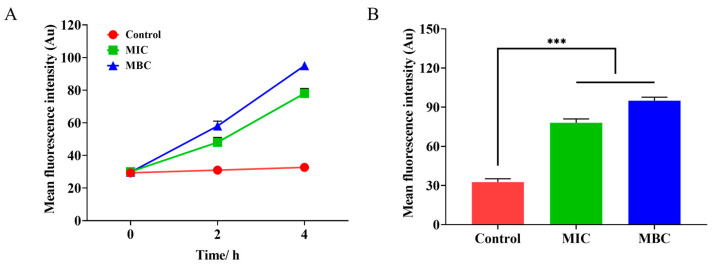
The depolarization state of S.Tm cells following treatment with varying concentrations of LCEO. (**A**) Trend of cell membrane potential. (**B**) The difference in membrane potential between different LCEO treatment groups and the control group at 4 h. *** *p* < 0.001 were considered significant.

The depolarization state of S.Tm cells following treatment with varying concentrations of LCEO is illustrated in Figure 4A. Compared to the control group, the fluorescence intensity in both the MIC and MBC groups was significantly elevated after 2 h of LCEO treatment. As the incubation time increased, the trend of cell membrane depolarization intensified further, peaking at 4 h. Figure 4B demonstrates that at 4 h, both the MIC and MBC groups exhibited extremely significant differences when compared to the control group.

#### 2.5.2. Nucleic Acid Leakage Analysis

**Figure 5:**
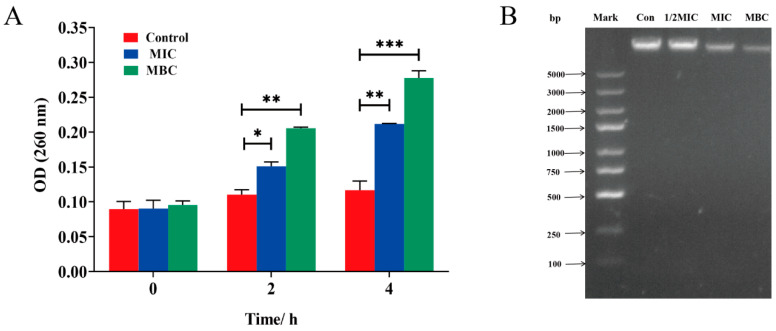
Effects of LCEO on nucleic acids in S.Tm cells. (**A**) Extracellular DNA content (OD_260_ nm). (**B**) Gel electrophoresis of intracellular DNA content. * *p* < 0.05, ** *p* < 0.01, and *** *p* < 0.001 were considered significant.

LCEO can increase the leakage of bacterial intracellular nucleic acid. As depicted in Figure 5A, MIC and MBC treatments both led to the leakage of intracellular nucleic acid in bacteria. At 0 h, no significant differences were observed between the blank control group and both the MIC and MBC groups, indicating the absence of nucleic acid leakage. However, at 2 h, nucleic acid leakage was detected in both the MIC and MBC groups, demonstrating significant differences when compared to the control group. After 4 h, the leakage situation worsened, with the values of the MIC and MBC groups increasing by 2.3 times and 2.9 times, respectively. To further describe the loss of nucleic acid, gel imaging technology was used to characterize the intracellular DNA content, as shown in Figure 5B. After 4 h of LCEO treatment, the DNA bands of MIC and MBC groups in the samples were significantly weakened, indicating a significant decrease in intracellular DNA content.

#### 2.5.3. Intracellular Protein Leakage

**Figure 6:**
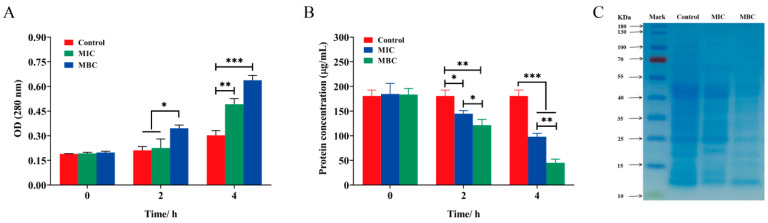
Effects of LCEO on proteins in S.Tm cells. (**A**) Extracellular protein content (OD_280_ nm). (**B**) Intracellular protein content, detected using a BCA protein quantification kit. (**C**) Gel electrophoresis of intracellular proteins. * *p* < 0.05, ** *p* < 0.01, and *** *p* < 0.001 were considered significant.

As shown in Figure 6A, both MBC and MIC treatments caused bacterial intracellular protein leakage. MIC leakage showed a significant difference from the control at 4 h, while MBC leakage showed a significant difference from the control at 2 h. The results showed that the effect of LCEO on bacterial intracellular nucleic acid and protein leakage was more pronounced at the concentration of MBC. As depicted in Figure 6B, further assessment of the changes in total protein content within S.Tm cells followed the LCEO treatment. It was observed that the protein content in S.Tm cells was significantly reduced compared to that in the control group, and with increasing LCEO concentration, a wider range of protein loss was observed. Electrophoresis results also reflect protein loss, as illustrated in Figure 6C. The SDS-PAGE pattern revealed distinct protein bands in the control group. After 4 h of LCEO treatment, the protein bands in the sample progressively weakened or even vanished. These findings suggest that LCEO treatment impacts intracellular protein synthesis in Salmonella cells.

### 2.6. Intracellular Enzyme Activity

**Figure 7:**
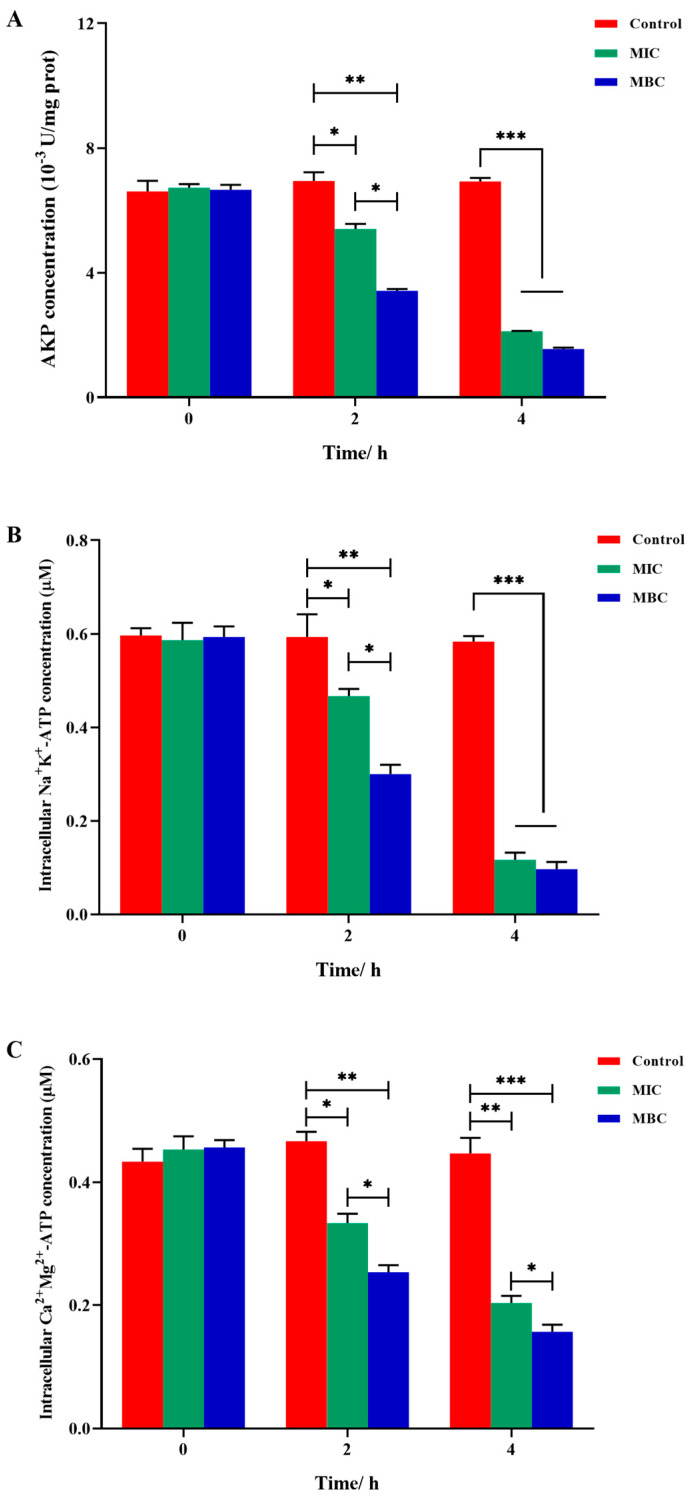
The effect of LCEO processing on S.Tm enzyme activity. (**A**) The effect of LCEO on AKP content. (**B**) The effect of LCEO on Na^+^K^+^-ATPase activity. (**C**) The effect of LCEO on Ca^2+^Mg^2+^-ATPase activity. * *p* < 0.05, ** *p* < 0.01, and *** *p* < 0.001 were considered significant.

The variations in AKP concentration are illustrated in Figure 7A. Initially, at the 0 h mark, no discernible difference in AKP content was observed between the control and experimental groups. Subsequent to 2 h of LCEO treatment, a notable decrease in AKP concentration was evident, with the MIC and MBC groups exhibiting reductions of 30.2% and 55.8%, respectively, in comparison to that in the control group. By the 4 h mark of LCEO treatment, the decline in AKP concentration intensified further, with the MIC and MBC groups showing decreases of 77.9% and 83.8%, respectively. Notably, the change in AKP concentration within the MBC group was more substantial when compared to that of the MIC group.

Figure 7B illustrates the effect of LCEO on the activity of S.Tm Na⁺K⁺-ATPase. Following 2 h of LCEO treatment, the Na^+^K^+^-ATPase content in the MIC and MBC groups showed decreases of 21.2% and 49.4%, respectively, with significant differences observed between these groups. After 4 h of treatment, while the Na^+^K^+^-ATPase content in both groups continued to decline, by 79.9% in the MIC group and 83.4% in the MBC group, the difference between these groups diminished, suggesting that over time, the MIC dose of LCEO could achieve a bactericidal effect comparable to that of the MBC dose. Figure 7C illustrates the effect of LCEO on the activity of S.Tm Ca^2+^Mg^2+^-ATPase. After 2 h of LCEO treatment, the MIC and MBC groups saw reductions of 28.7% and 45.8%, respectively. By the 4 h mark, these reductions widened further, with the Ca^2+^Mg^2+^-ATPase content decreasing by 54.6% in the MIC group and by 46.9% in the MBC group compared to that of the control.

### 2.7. Effect of LCEO on S.Tm Biofilm

**Figure 8:**
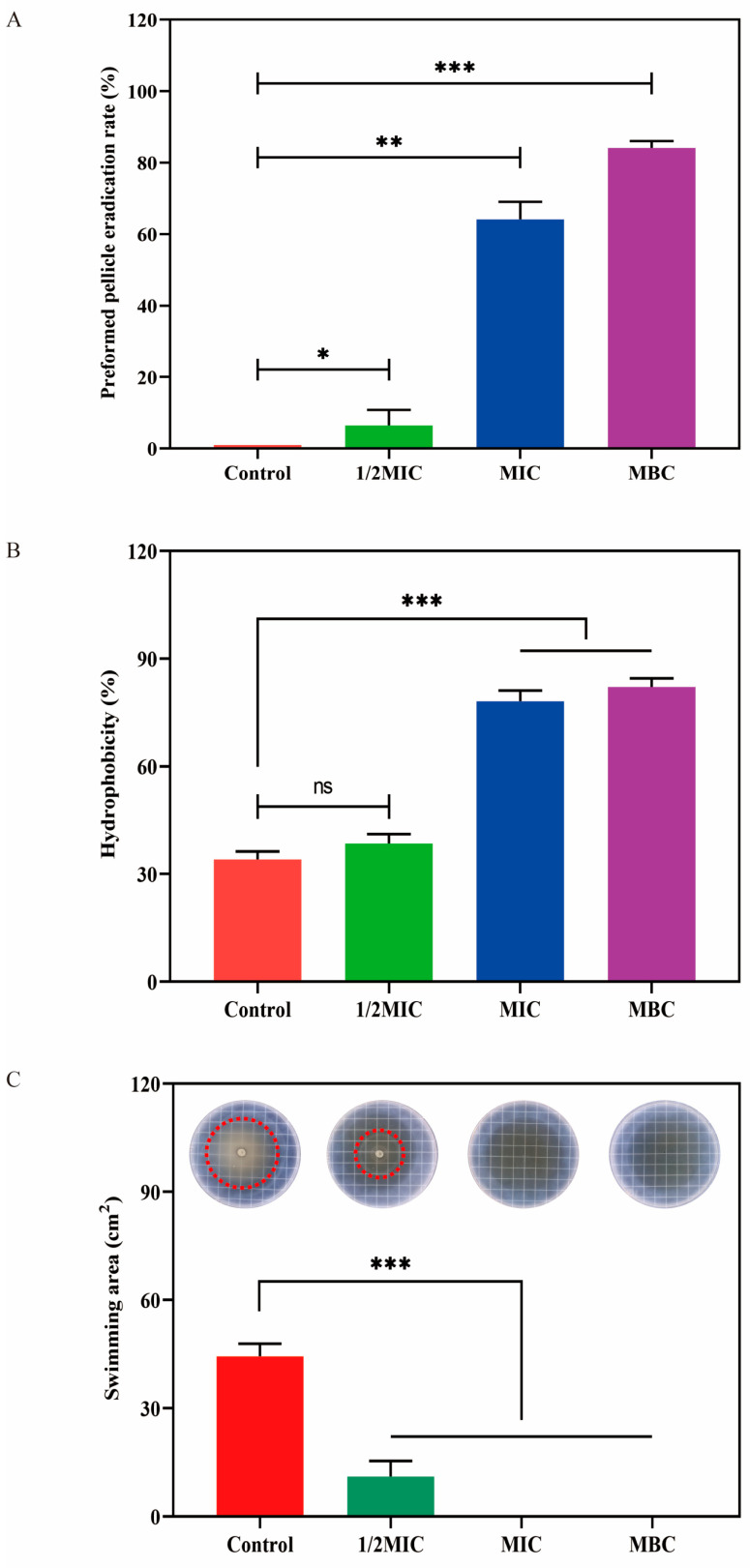
Effect of LCEO on biofilm formation ability. (**A**) The biofilm clearance effect of LCEO on S.Tm. (**B**) Cell surface hydrophobicity. (**C**) The swimming motility of S.Tm (The red dotted areas represent the swimming area of S.Tm). * *p* < 0.05, ** *p* < 0.01, and *** *p* < 0.001 were considered significant.

Figure 8A shows that cells treated with LCEO exhibited significant signs of biofilm clearance compared to the control cells. Notably, even at a concentration as low as 1/2 MIC, the introduction of LCEO led to a biofilm clearance rate of 6.5%, marking a significant divergence from the control group. As the concentration escalated to the MIC level, the reduction in biofilm became particularly pronounced, with the clearance rate to 64.2%. Furthermore, when the concentration of the LCEO reached the MBC, the removal efficacy of biofilms approached its zenith, achieving a clearance rate as impressive as 84.1%, leaving the biofilms nearly eradicated.

Figure 8B demonstrates that subsequent to LCEO treatment, the hydrophobicity of the S.Tm cell surface underwent a marked enhancement. A distinct correlation emerged between this surge in hydrophobicity and the concentration of essential oil utilized. Notably, after 1/2 MIC treatment, the hydrophobicity of the cell surface remained largely comparable to that of the control group, recording a value of 38.5% versus the control’s 34.1%. However, when the cells were treated with the MIC and MBC of LCEO, the hydrophobicity of the cell surface rose to 78.1% and 82.1%, respectively, exhibiting significant differences.

The swimming motility of S.Tm was assessed under varying concentrations of LCEO, with the results presented in Figure 8C. In the control group, the swimming area of the cells measured a substantial 14.1 cm^2^. However, upon exposure to 1/2 MIC of LCEO, this area decreased dramatically to merely 3.5 cm^2^. Furthermore, when the concentration of LCEO was elevated to the MIC level, swimming motility was completely suppressed. An analogous trend was observed following MBC treatment, yielding a complete inhibition rate of 100%.

### 2.8. The Antibacterial Activity of LCEO Chemical Components Against S.Tm

LCEO primarily comprises several potential antimicrobial substances, including citral (a mixture of geranial and neral), α-terpineol, and ylangene. Consequently, the antimicrobial effects of these compounds against S.Tm were further validated. As shown in Table 3, the DIZ values for citral, α-terpineol, and ylangene against S.Tm were 30.7 ± 0.7 mm, 0 mm, and 1.1 ± 0.2 mm, respectively. This indicates that citral and ylangene exhibit antibacterial effects against S.Tm, while α-terpineol does not. Additionally, MIC and MBC tests yielded similar results. The MIC and MBC values for citral were 0.20 mg/mL and 0.40 mg/mL, respectively, demonstrating a slightly superior antimicrobial effect compared to that of LCEO. Neither ylangene nor α-terpineol displayed detectable MIC and MBC values, indicating that while ylangene possesses certain antibacterial properties, it exhibits poor bactericidal effects. In contrast, α-terpineol showed no antibacterial activity.

## 3. Discussion

Food safety issues arising from foodborne pathogens present a significant challenge to global public health. While traditional chemically synthesized antimicrobial agents are effective, concerns over potential resistance development and high residue levels have prompted researchers to explore natural plant-based antimicrobials [9]. *Litsea cubeba* essential oil, a plant secondary metabolite with broad-spectrum biological activities, has received considerable attention for its antimicrobial properties, primarily due to its main component, citral [11]. However, existing research has yet to systematically analyze the regional variations in the chemical composition of this essential oil and its multifaceted antimicrobial mechanisms, which impedes its industrial application. This study employed GC–MS to systematically analyze the chemical components of *Litsea cubeba* essential oil and elucidates its antimicrobial mechanisms through various pathways, including disruption of cell membrane integrity, interference with macromolecular functions, and inhibition of biofilm formation. The findings of this research aim to provide theoretical support and technical references for the development of natural essential oil-based food preservatives and novel antimicrobial agents.

This study analyzed the chemical composition of LCEO using GC–MS, revealing that its primary components are neral (29.76%) and geranial (35.62%), which together account for 65.38% of the total composition. These findings are consistent with previous studies that report citral (a mixture of neral and geranial), as the predominant component of LCEO [18,19]. However, the minor components identified in this study show certain discrepancies compared to those documented in other literature. For instance, this experiment detected α-terpineol (6.25%), ylangene (4.38%), geraniol (3.43%), and α-pinene (3.18%), while Duc et al. [20] reported higher concentrations of caryophyllene oxide (11.0%), cis-β-guaiene (6.4%), khusimone (3.9%), and bicycloelemene (3.7%). Another report has indicated that the minor components present in higher concentrations in LCEO are eucalyptol (16.8%), linalool (10.1%), 4-methyl-cyclohex-3-en-1-ol (8.7%), and cyclohex-3-en-1-ylmethane (6.94%). Previous studies have indicated that aldehydes and alcohols present in essential oils are the primary active antimicrobial substances. These differences may arise from variations in geographical origin, harvest time and location, climate, and other factors [21,22]. In this study, despite the variations in the minor components of LCEO, the consistency of its major components provides compelling evidence for its potential antimicrobial activity [20,23,24].

This study demonstrated that LCEO exhibited antimicrobial activity against all four foodborne bacteria tested, with the most pronounced antibacterial effect observed against S.Tm and the least against *Bacillus cereus*. These findings align with those of Yang et al. [25], suggesting that LCEO exhibits superior antibacterial activity against Gram-negative bacteria compared to that against Gram-positive bacteria. This phenomenon may be attributed to the hydrophobic active compounds in LCEO, which are more likely to adhere to the cell surface of Gram-negative bacteria and penetrate the intracellular space via target sites such as the plasma membrane and membrane-bound enzymes. This interaction leads to cell membrane damage and the leakage of intracellular contents [26,27]. The MIC and MBC values are widely recognized as qualitative and quantitative indicators for assessing the antimicrobial activity of essential oils. In this study, the MIC value of LCEO against S.Tm was significantly lower than those reported for other essential oils [13,14], indicating that LCEO possesses substantial antibacterial activity against S.Tm.

The volatile nature and poor stability of essential oils limit their application scope. This study demonstrated that temperature and pH significantly influenced the antimicrobial activity of LCEO, while ultraviolet light did not have an impact, thus providing a theoretical basis for the application conditions of LCEO. Rahman et al. [28] found that citral maintained high antimicrobial activity even after 24 h of ultraviolet light exposure, indicating that citral exhibits a certain degree of stability under light conditions. This finding aligns with the results of the current study, which showed that the antimicrobial activity of LCEO did not significantly change after short-term ultraviolet light treatment. Temperature is a critical factor affecting the stability of essential oils, as the volatile components can evaporate or decompose at elevated temperatures [29,30]. Hąc-Wydro et al. [31] discovered that limonene exhibits greater efficacy against model bacterial membranes at lower temperatures. They noted that higher temperatures promote the evaporation of limonene, thereby reducing its concentration and antibacterial activity. This observation is consistent with the antibacterial results of LCEO at varying temperatures in this study, suggesting that the volatile compounds in LCEO are more stable at lower temperatures, leading to enhanced antibacterial effects compared to those at higher temperatures [26,32]. pH is a critical factor influencing the stability of essential oils and bacterial growth. Previous studies have shown that both an acidic environment with pH ≤ 4 and an alkaline environment with pH ≥ 9 can affect the growth of S.Tm [33,34]. The results of this study align with those from the existing literature, as inhibition zones were observed at pH 4 and pH 9; however, DIZ < 8, leading to the conclusion that they lacked antibacterial activity [5]. Furthermore, Fernando et al. [35] assessed the inhibitory effects of essential oils against *Escherichia coli* and S.Tm at varying pH levels (5.0, 5.5, or 6.0) using the viable cell count method, concluding that antibacterial activity diminished as pH decreased. A study conducted by Guo et al. [24] demonstrated that the antibacterial activity of amomum essential oil against *Staphylococcus aureus* diminishes as pH increases (6.0, 8.0, 10.0, and 12.0). Similarly, our research indicates that variations in pH influenced the antibacterial efficacy of LCEO against S.Tm. This phenomenon may be attributed to the decomposition or chemical reactions of the terpenoid antibacterial compounds present in LCEO [36] under strongly acidic or alkaline conditions. The findings of this study provide a theoretical foundation for the further development and application of LCEO.

The cell membrane and cell wall function as protective barriers essential for the survival and integrity of bacteria. When these barriers are compromised, macromolecules such as proteins and nucleic acids can leak out of the cell, resulting in metabolic dysfunction and, ultimately, bacterial death [13]. SEM and PI imaging revealed significant morphological changes in S.Tm following treatment with LCEO, confirming severe damage to the membrane structure and cytoplasmic leakage. This suggests that LCEO can penetrate the cell, interfering with normal physiological processes and thereby inhibiting bacterial growth. The findings of this study align with previous reports [37,38], demonstrating that LCEO disrupts the integrity of the S.Tm cell membrane, preventing the maintenance of normal physiological metabolic activities and ultimately leading to bacterial death.

To further elucidate the changes in S.Tm cells treated with LCEO, this study selected three crucial biomacromolecules vital for cell survival as targets: DNA, total soluble proteins, and ATP. These biomacromolecules correspond to genetic information, energy sources, and catalysts (enzymes) within the cell, respectively [38]. Our research indicates that LCEO inhibited bacterial growth by interfering with the expression of S.Tm DNA, a process potentially mediated by its main component, citral. Citral can exacerbate the oxidative stress response in S.Tm cells, leading to damage in DNA, lipids, and proteins, and inducing an adaptive response characterized by ATP depletion within the cells [39]. When DNA is cleaved by small molecules, particularly through oxidation, the phosphodiester bonds in DNA are disrupted, blocking the normal physiological function of DNA, which inhibits cell growth or even results in cell death [40]. Proteins are essential biomolecules for bacterial survival, widely present in the cell membrane and cytoplasm [41,42]. Song et al. [13] analyzed the total soluble proteins of Staphylococcus aureus exposed to citrus essential oil using SDS-PAGE and found that citrus essential oil can inhibit protein release or disrupt the synthesis of intracellular proteins, thereby suppressing bacterial activity. This finding is similar to the loss of total cellular protein observed after LCEO treatment in this study. Therefore, it can be concluded that LCEO not only disrupts the integrity of the cell membrane, leading to the leakage of intracellular components, but may also inhibit protein synthesis, thereby killing bacterial cells.

ATP plays a crucial role in energy storage and the transmembrane transport of substances. Na^+^K^+^-ATPase is primarily located in the cell membrane and utilizes ATP to transport sodium ions across the membrane, exchanging them for potassium ions outside the cell. This process counteracts the concentration gradient and maintains cell membrane permeability [43]. An increase in intracellular calcium ion concentration can induce apoptosis. Ca^2+^Mg^2+^-ATPase catalyzes the hydrolysis of ATP within the cell, transporting Ca^2+^ to the extracellular space, which is essential for maintaining a low intracellular calcium ion concentration and ensuring cell viability [44]. This study demonstrates that after treatment with LCEO, the levels of Na^+^K^+^-ATPase and Ca^2+^Mg^2+^-ATPase in *S*.*Tm* significantly decreased, and this reduction was dependent on the concentration and duration of LCEO exposure. This decrease may be attributed to the inhibition of Na^+^K^+^-ATPase and Ca^2+^Mg^2+^-ATPase activity following LCEO treatment, which disrupts the intracellular transport of Na^+^ and Ca^2+^, ultimately leading to bacterial death.

The complex formation mechanism of bacterial biofilms involves not only the motility of the bacteria themselves but is also profoundly influenced by their surface characteristics, which play a crucial role in the adhesion and aggregation processes between bacteria and interfaces [45]. Studies have shown that reducing the hydrophobicity of the cell surface significantly affects bacterial adhesion and aggregation on interfaces, thereby slowing down the biofilm formation process [46,47]. Our results indicate that LCEO has a significant inhibitory effect on the formation of S.Tm bacterial biofilms; however, LCEO did not reduce but rather increased the hydrophobicity of the cell surface. This phenomenon may be attributed to the enhanced impact of LCEO on the permeability of the cell wall and membrane, leading to the continuous leakage of intracellular components such as proteins and lipids. These natural surfactants effectively reduce the hydrophobicity of cell surfaces, fundamentally altering the adhesive properties of the cells [1,2]. This finding is consistent with the results of Liu et al. [48] in their study of Listeria monocytogenes in garlic essential oil.

This study also evaluated the antimicrobial capabilities of the components in LCEO. The results indicated that citral, the primary component of LCEO, exhibited strong antibacterial activity, with MIC and MBC values of 0.20 mg/mL and 0.40 mg/mL, respectively, significantly outperforming other essential oils. Although ylangene demonstrated some antibacterial effects, its bactericidal efficacy was limited, likely due to its low concentration, which did not reach the required bactericidal threshold [49]. Furthermore, existing research suggests that α-terpineol primarily exhibits anti-inflammatory and antioxidant activities, with relatively low antibacterial effects [50,51]. This study investigated the antimicrobial activities of several individual compounds and identified those with notable antimicrobial properties, although their conformational relationships require further investigation.

## 4. Materials and Methods

### 4.1. Materials and Reagents

The four foodborne pathogens were *Escherichia coli* ATCC 25922, S.Tm ATCC14028, *Listeria monocytogenes* ATCC 19115, and *Bacillus cereus* ATCC 12022, all of which were purchased from the China Industrial Microbial Culture Collection Center (Beijing, China). *Litsea cubeba* essential oil was purchased from Jiangxi Xin Sen Natural Plant Oil Co., Ltd. (Ji’an, China). The LCEO solvent containing 1% dimethyl sulfoxide and 0.1% Tween 80 was prepared and stored in a brown glass vial and was disinfected before use.

### 4.2. LCEO Compound Analysis

The compound LCEO was diluted with cyclohexane to achieve a concentration of 1% (*v*/*v*) and subsequently analyzed using a QP-2010 Ultra system (Shimadzu, Tokyo, Japan). The gas chromatography–mass spectrometry (GC–MS) analysis was conducted following previously established methods [52]. An HP-5MS capillary column was employed for separation. The temperature program commenced at 50 °C for 2 min, followed by a gradual increase at a rate of 5 °C/min to 240 °C, where it was held for an additional 14 min. The column pressure was set at 50 kPa, with He (99.99%) serving as the carrier gas at a flow rate of 1.0 mL/min. The inlet temperature was maintained at 250 °C, and the split ratio was configured at 1:50. The relative percentages of each component within LCEO were determined through area normalization. The utilized search databases included the NIST 17 MS Library.

### 4.3. Antibacterial Activity

#### 4.3.1. Agar Paper Diffusion Method

The inhibitory effect of LCEO on bacteria was assessed using the agar diffusion method [53]. In brief, 100 μL of bacterial suspension (10^8^ CFU/mL) was added to the culture dish. A sterile filter paper disk, 6 mm in diameter, was then placed in the center of the agar, followed by the addition of 5 μL of LCEO. The blank solution without LCEO was used as a negative control. *Berberine hydrochloride* (5 mg/disc, from National Institutes for Food and Drug Contro, Beijing, China) was used as a positive control against the foodborne pathogens tested. The dishes were incubated at 37 °C for 24 h, after which the diameter of the inhibitory zone (DIZ) was measured.

#### 4.3.2. Determination of MIC and MBC

The MIC and MBC of LCEO against S.Tm were determined using a double dilution method. LCEO was initially dissolved in sterile LB broth containing 1% DMSO and 0.1% Tween 80 at a concentration of 25.6 mg/mL. This solution was subsequently diluted to achieve a final concentration range of 0.10–12.8 mg/mL (0.1, 0.2, 0.4, 0.8, 1.6, 3.2, 6.4, and 12.8) in a 10 mL sterile test tube. A similar volume of bacterial suspension (10^7^ CFU/mL) was co-cultured with the LCEO solution on a 96-well microplate and incubated at 37 °C for 24 h. The change in absorbance at 600 nm was measured using an enzyme-linked immunosorbent assay (ELISA) reader to evaluate bacterial growth. The blank solution without LCEO was used as a negative control. *Berberine hydrochloride* was used as a positive control, with a final concentration range of 0.15–19.2 mg/mL (0.15, 0.3, 0.6, 1.2, 2.4, 4.8, 9.6, and 19.2). Following the MIC determination, the liquid from the wells exhibiting no significant growth was diluted 100-fold with sterile LB broth and transferred to a new 96-well microplate for further incubation at 37 °C for 24 h. The minimum concentration of LCEO that resulted in no significant growth was defined as the MBC.

#### 4.3.3. Time–Killing Curve

Referring to the experimental method of Wang et al. [17], with slight modifications, the time–killing curve was determined using the agar plate viable count method to investigate the inhibitory effect of various concentrations of LCEO (1/2 MIC, MIC, MBC) on the growth of S.Tm. LCEO was mixed with a logarithmic growth phase suspension of S.Tm bacteria (10^7^ CFU/mL) at varying concentrations, while the group without LCEO served as the blank control. Incubation was conducted at 37 °C with shaking at 160 rpm for 0, 1, 2, 4, 6, 12, and 24 h. The mixed solution was diluted, and 100 µL was evenly spread onto LB agar plates. The plates were incubated at 37 °C for 24 h, followed by colony counting. The time–killing curve was plotted with time on the x-axis and log10 (CFU/mL) on the y-axis. Each experiment was repeated three times.

### 4.4. Study on Antibacterial Stability of LCEO

#### 4.4.1. Effect of Temperature on the Antibacterial Activity of LCEO

Following the experimental method outlined by Guo et al. [24], with slight modifications, LCEO was heated to temperatures of 20, 30, 40, 60, 80, and 100 °C for a duration of 30 min, after which it was allowed to cool naturally to room temperature. Antibacterial activity was assessed using the agar diffusion method described in Section 4.3.1, with untreated LCEO serving as the control (at room temperature). The antibacterial activity of LCEO was determined using the DIZ. All experiments were repeated three times.

#### 4.4.2. Effect of Light Exposure on the Antibacterial Activity of LCEO

LCEO was exposed to a 254 nm UV lamp for durations of 0, 30, 60, 90, 120, and 180 min. Then, as previously described, antibacterial activity was measured using the agar disk method, with LCEO that was not exposed to ultraviolet radiation serving as the blank control. All experiments were repeated three times.

#### 4.4.3. The Effect of pH on the Antibacterial Activity of LCEO

LCEO was adjusted to pH 2.0, 4.0, 6.0, 8.0, 10.0, and 12.0 using a HCl solution (1.0 mol/L) and a NaOH solution (1.0 mol/L), respectively. As previously mentioned, the antibacterial activity was evaluated using the agar disk method, with unadjusted LCEO serving as the control. Additionally, a negative control (solution without essential oils at different pH) was established to eliminate potential interference. All experiments were conducted three times.

### 4.5. Cell Morphology and Membrane Damage

#### 4.5.1. Morphological Analysis Using Scanning Electron Microscopy (SEM)

The morphological analysis of S.Tm was conducted using SEM. A bacterial suspension of S.Tm in the logarithmic growth phase was prepared, to which LCEO was added at a final concentration equal to the MIC. The S.Tm bacterial suspension in the blank control group was left untreated. Both sets of bacterial solutions were incubated in a shaker at a constant temperature of 37 °C with a rotation speed of 160 rpm for 4 h. Following incubation, the samples were centrifuged at 5000 rpm for 5 min, washed twice with PBS, and fixed with glutaraldehyde at a concentration of 2.5% (*v*/*v*). The samples were then stored at 4 °C overnight. Subsequently, the samples underwent dehydration using sequential concentrations of ethanol: 30%, 50%, 70%, 90%, and 100%. Finally, the samples were subjected to cathodic gold sputtering, and the morphology of the bacterial cells was observed under SEM at an acceleration voltage of 5.0 kV.

#### 4.5.2. Membrane Damage Assessment

The MIC and MBC of LCEO were incorporated into the medium, and untreated samples were used as controls. A previously established method was used with some modifications [54]. The logarithmic growth phase bacterial culture was centrifuged at 5000 rpm for 10 min, followed by two washes with PBS and subsequent dilution of the bacterial solution to a concentration of 10^6^ CFU/mL. The bacterial solution was then incubated with a PI staining solution in the dark at 37 °C for 30 min. Stained cells were extracted (5 μL) and observed under a fluorescence microscope (Leica Microsystems, Wetzlar, Germany) with an excitation wavelength of 535 nm and an emission wavelength of 615 nm.

### 4.6. Research on Antibacterial Mechanisms

#### 4.6.1. Membrane Potential Analysis

The effect of LCEO on the membrane potential of S.Tm was investigated using the membrane potential fluorescence probe DiBAC4(3) (bis-(1,3-Dibarbituric acid)-Trimethine oxanol, Solarbio, Beijing, China) [55]. A suspension of S.Tm bacteria (10^7^ CFU/mL) was treated with various concentrations of LCEO (MIC and MBC) for durations of 0, 2, and 4 h, with untreated samples serving as controls. Samples were collected for analysis at 0, 2, and 4 h. Briefly, after washing the samples with PBS, they were centrifuged at 4000 rpm for 5 min. The samples were then resuspended in PBS, and DiBAC4(3) was added to achieve a final concentration of 1 µM. The mixture was thoroughly mixed and incubated in the dark at 37 °C for 30 min. Subsequently, 200 µL of the cell sample was transferred to a 96-well plate for detection, using an excitation wavelength of 516 nm and an emission wavelength of 490 nm. The experiment was performed in triplicate, with two independent replicates.

#### 4.6.2. Determination of Extracellular Nucleic Acid and Protein

To assess cell integrity, extracellular nucleic acids and proteins were evaluated. A suspension of S.Tm bacteria (10^7^ CFU/mL) was treated with various concentrations of LCEO (MIC and MBC) for durations of 0, 2, and 4 h, respectively, with untreated samples serving as controls. Following treatment, the cell suspension was centrifuged at 7340 rpm for 10 min, and the supernatant was filtered through a 0.22 μm microporous membrane. The absorbance at OD_260_ nm and OD_280_ nm was measured using a microplate reader to quantify the nucleic acids and proteins released from the bacterial cells. Each experiment was conducted in triplicate.

The precipitated cells were collected after centrifugation, washed three times with PBS, and then re-suspended. Total DNA from S.Tm was extracted using a total DNA extraction kit (Solarbio). The extracted DNA was confirmed to have a ratio of 260 nm to 280 nm (A260/A280 > 1.80), indicating the absence of protein contamination. DNA fragments were isolated by electrophoresis in a 0.8% agarose gel at 130 V and stained for 40 min.

#### 4.6.3. BCA Protein Quantification Test Kit for Determining Protein Content

A bacterial suspension in the logarithmic growth phase was taken, centrifuged, and resuspended in PBS to OD_600_ = 2.0. Subsequently, LCEO was added to the final concentrations corresponding to the MIC and MBC, with untreated samples serving as controls. All samples were incubated at 37 °C for 4 h. After incubation, 1 mL of the bacterial solution was taken, centrifuged at 8040 rpm for 10 min at 4 °C, and processed using an ultrasonic cell disruption system. The sample was centrifuged again to extract the supernatant, which was kept on ice for subsequent testing. The absorbance at 562 nm was measured using enzyme-linked immunosorbent assay (ELISA) following the protocol outlined in the BCA protein assay kit, and the values were recorded. Cell proteins were analyzed via sodium dodecyl sulfate polyacrylamide gel electrophoresis (SDS-PAGE). The samples were combined with a 5-fold volume of protein loading buffer, boiled for 5 min, then cooled and centrifuged. The gel was prepared using the SDS-PAGE gel preparation kit (Solarbio, Beijing, China), consisting of a 5% stacking gel and a 12% separating gel. After electrophoresis, the gel was stained with Coomassie Brilliant Blue R-250 (Solarbio, Beijing, China) and subjected to decolorization analysis.

### 4.7. Effect of LCEO on Bacterial Enzyme Activity

#### 4.7.1. Determination of Alkaline Phosphatase (AKP) Activity

According to the methodology outlined in the alkaline phosphatase (AKP) assay kit, the pretreatment of cells mirrors that employed for protein quantification. In summary, the bacterial suspension, treated with various concentrations of LCEO, was centrifuged to isolate the precipitated bacterial cells. Subsequently, alkaline phosphatase extract was added to the cells, ensuring thorough mixing as per the kit instructions. The supernatant was then collected via centrifugation and placed on ice for subsequent measurements. Following the AKP reagent kit protocol, the absorbance was measured at 520 nm using an enzyme-linked immunosorbent assay (ELISA) reader, and the corresponding value was recorded.

#### 4.7.2. Content and Activity of Adenosine Triphosphate (ATP)

The S.Tm bacterial suspension (10^7^ CFU/mL) was treated with varying concentrations of LCEO, categorized into the MIC group, MBC group, and control group. The alterations in the levels of Na^+^K^+^-ATPase and Ca^2+^Mg^2+^-ATPase within the bacterial cells were assessed using an ATPase activity assay kit (Sangon Biotech, Shanghai, China) at time points of 0, 2, and 4 h.

### 4.8. Antibiofilm Activity of LCEO

#### 4.8.1. Biofilm Clearance Rate

The crystal violet staining method was employed to assess the biofilm clearance rate [56]. Initially, 200 μL of methanol was added to the biofilm treated with varying concentrations of LCEO and allowed to fix for 15 min. After the methanol was removed, 200 μL of a 0.01 mol/L crystal violet solution was introduced for staining, lasting 20 min. Following this, the crystal violet solution was discarded, and 200 μL of 33% glacial acetic acid was added. One hour later, the OD_600_ value of the sample was measured, with each sample being replicated three times. The biofilm clearance rate was calculated using Formula (1).

(1) $$Biofilm\;clearance\;rate\;(\%)=\frac{A_{0}-A_{EO}}{A_{0}}\times 100\%$$

In the formula, A_0_ is the absorbance value of the control group and A_EO_ is the absorbance value of the essential oil treatment group.

#### 4.8.2. Determination of Bacterial Surface Hydrophobicity

The hydrophobicity of the bacterial surface was determined using the microbial adhesion to hydrocarbons method [57]. Logarithmic-phase S.Tm cells were washed three times with PBS and then suspended to OD_600_ = 0.5. A 3 mL bacterial suspension was added to 3 mL PBS, achieving final concentrations of essential oil at 1/2 MIC, MIC, and MBC, respectively. A control group without essential oil was also prepared, and samples were taken after standing at 37 °C for 4 h. First, the OD_600_ value (A_1_) of the bacterial solution was measured. Then, 3 mL of the aforementioned bacterial solution was pipetted and mixed with 400 μL of n-hexadecane. The mixture was vortexed for 1 min and allowed to stand at 37 °C for 15 min. Subsequently, the lower aqueous phase was removed, and its OD_600_ value (A_2_) was measured. Finally, surface hydrophobicity was calculated according to Formula (2).

(2) $$Cell\;surface\;hydrophobicity\;(\%)=\frac{A_{1}-A_{2}}{A_{1}}\times 100\%$$

In the formula: A_1_ was the value of the bacterial solution; A_2_ was the value of the lower aqueous phase.

#### 4.8.3. Cell Swimming Ability Analysis

In accordance with the preparation method for the swimming medium plate, swimming media were prepared with LCEO at concentrations of 1/2 MIC, MIC, and MBC, respectively. A medium without LCEO served as the blank control. A volume of 5 µL of bacterial droplets, with a cell concentration of 10^6^ CFU/mL, was absorbed and placed at the center of the petri dish. After the droplets infiltrated the medium, they were cultured at 37 °C for 24 h. The diameter of the bacterial swimming ring was measured using the cross-crossing method, and the area of the swimming ring was subsequently calculated.

### 4.9. Antibacterial Activity of Chemical Components in LCEO

The IZD of citral (a mixture of geranial and neral), α-terpineol, and ylangene at a concentration of 20 mg/mL was measured to identify compounds exhibiting antibacterial activity. This activity was further quantified in subsequent experiments to determine their MIC and MBC values. The specific experimental methodology employed remained consistent with that described in Section 4.3.

### 4.10. Data Analysis

All measurements were repeated and the results were the average of three independent experiments. A one-way analysis of variance (ANOVA) was conducted using SPSS Statistics 21 software. The data were presented as the mean ± standard deviation (SD) of at least three independent experiments. * *p* < 0.05, ** *p* < 0.01, and *** *p* < 0.001 were considered significant.

All measurements were conducted in triplicate, and the results are expressed as the mean of three independent experiments. A one-way analysis of variance (ANOVA) was performed using SPSS Statistics 21 software. The data are presented as the mean ± standard deviation (SD) from at least three independent experiments. Statistical significance was defined as *p < 0.05, ** *p* < 0.01, and *** *p* < 0.001.

## 5. Conclusions

In summary, this study employed a multidimensional analysis to elucidate the synergistic antibacterial mechanisms of LCEO against foodborne pathogens. It confirmed that LCEO exerted its antibacterial activity by disrupting the permeability barrier of the outer membrane in S.Tm, which led to intracellular ATP depletion, ion homeostasis imbalance, and macromolecular damage. Furthermore, the study demonstrated that the antibacterial activity of the essential oil was sensitive to environmental factors. Consequently, the development of sustained-release encapsulation technology or combination systems with other antibacterial agents is crucial for overcoming its volatility limitations. The findings not only enrich the theoretical framework of antibacterial plant essential oils at the molecular level but also provide a scientific basis for the development of natural food preservatives and disinfectants for feed additives based on LCEO.

## Figures and Tables

**Table 1:** Chemical composition of *Litsea cubeba* essential oil (%).

Number	RT (min)	RI	Compounds	PA (%)
1	7.04	968	α-pinene	3.18
2	7.94	989	α-phellandrene	2.76
3	8.36	999	6-methyl-5-hepten-2-one	1.56
4	12.24	1088	α-terpinene	1.01
5	15.03	1153	citronellal	2.11
6	16.32	1183	isocitral	0.55
7	16.59	1189	α-terpineol	6.25
8	17.79	1219	*cis*-isopiperitenol	0.53
9	18.23	1231	nerol	1.79
10	18.67	1243	neral	29.76
11	19.25	1258	geraniol	3.43
12	19.79	1273	geranial	35.62
13	21.68	1328	2,6,11-Trimethyldodecane	0.60
14	22.01	1338	γ-elemene	0.71
15	22.41	1351	α-terpinyl acetate	0.42
16	23.21	1377	ylangene	4.38
17	23.51	1386	lavandulyl acetate	0.47
18	23.71	1393	β-elemene	0.21
19	25.56	1459	β-famesene	0.93
20	26.93	1510	α-bisabolene	2.85
21	27.30	1525	δ-cadinene	0.36

Abbreviations: RT, retention time; RI, retention indices; PA, relative peak area.

**Table 2:** The DIZ, MIC, and MBC values of LCEO and berberine hydrochloride against the tested bacteria.

Germs	DIZ (mm)		MIC (mg/ mL)		MBC (mg/ mL)	
	Bh	LCEO	Bh	LCEO	Bh	LCEO
*E*. *coli*	19.0 ± 0.1	22.1 ± 0.5	0.6	0.8	1.2	1.2
S. Tm	19.9 ± 0.3	25.5 ± 0.7	0.6	0.4	1.2	0.8
L. m	16.6 ± 0.3	20.2 ± 1.2	1.2	0.8	2.4	1.6
B. c	17.2 ± 0.2	18.9 ± 0.5	1.2	0.8	2.4	2.0

Abbreviations: *E. coli*, *Escherichia coli*; S.Tm, *Salmonella typhimurium*; L.m, *Listeria monocytogenes*; B. c, *Bacillus cereus*; Bh, berberine hydrochloride; LCEO, *Litsea cubeba* essential oil.

**Table 3:** The DIZ, MIC, and MBC of LCEO chemical components composition against S.Tm.

Components	DIZ (mm)	MIC (mg/mL)	MBC (mg/mL)
citral	30.7 ± 0.7	0.2	0.4
α-terpineol	0	-	-
ylangene	1.1 ± 0.2	-	-

Note: citral (a mixture of geranial and neral). “-” indicates its poor antibacterial activity, and the MIC (MBC) values were not determined.

## Data Availability

The original contributions presented in this study are included in the article. Further inquiries can be directed to the corresponding author(s).

## References

[1] Garvey M. (2023). Foodborne Clostridioides Species: Pathogenicity, Virulence and Biocontrol Options. Microorganisms.

[2] Alvarado-Martinez Z., Julianingsih D., Tabashsum Z., Aditya A., Tung C., Phung A., Suh G., Hshieh K., Wall M., Kapadia S. (2023). Assessment of the prevalence, serotype, and antibiotic resistance pattern of *Salmonella enterica* in integrated farming systems in the Maryland-DC area. Front. Microbiol..

[3] Tran H.M., Prathan R., Hein S.T., Chuanchuen R. (2024). Microbiological Quality and Antimicrobial Resistance of Commercial Probiotic Products for Food-Producing Animals. Antibiotics.

[4] Calvigioni M., Cara A., Celandroni F., Mazzantini D., Panattoni A., Tirloni E., Bernardi C., Pinotti L., Stella S., Ghelardi E. (2022). Characterization of a *Bacillus cereus* strain associated with a large feed-related outbreak of severe infection in pigs. J. Appl. Microbiol..

[5] Finn L., Onyeaka H.O., Neill S. (2023). *Listeria monocytogenes* Biofilms in Food-Associated Environments: A Persistent Enigma. Foods.

[6] Lamas A., Miranda J.M., Regal P., Vázquez B., Franco C.M., Cepeda A. (2018). A comprehensive review of non-enterica subspecies of *Salmonella enterica*. Microbiol. Res..

[7] Zou G., Matuszewska M., Jia M., Zhou J., Ba X., Duan J., Zhang C., Zhao J., Tao M., Fan J. (2022). A Survey of Chinese Pig Farms and Human Healthcare Isolates Reveals Separate Human and Animal Methicillin-Resistant *Staphylococcus aureus* Populations. Adv. Sci..

[8] Majowicz S.E., Musto J., Scallan E., Angulo F.J., Kirk M., O’Brien S.J., Jones T.F., Fazil A., Hoekstra R.M. (2010). The Global Burden of Nontyphoidal *Salmonella* Gastroenteritis. Clin. Infect. Dis..

[9] de la Fuente-Nunez C., Cesaro A., Hancock R.E.W. (2023). Antibiotic failure: Beyond antimicrobial resistance. Drug Resist. Update.

[10] Huang X., Teng Z., Xie F., Wang G., Li Y., Liu X., Li S. (2024). Loading of cinnamon essential oil into electrospun octenylsuccinylated starch-pullulan nanofiber mats: Electrospinnability evaluation, structural characterization, and antibacterial potential. Food Hydrocolloid.

[11] Zheng K., Li W., Fu B., Fu M., Ren Q., Yang F., Qin C. (2018). Physical, antibacterial and antioxidant properties of chitosan films containing hardleaf oatchestnut starch and *Litsea cubeba* oil. Int. J. Biol. Macromol..

[12] Lucas-González R., Yilmaz B., Mousavi Khaneghah A., Hano C., Shariati M.A., Bangar S.P., Goksen G., Dhama K., Lorenzo J.M. (2023). Cinnamon: An antimicrobial ingredient for active packaging. Food Packag. Shelf.

[13] Song X., Liu T., Wang L., Liu L., Li X., Wu X. (2020). Antibacterial Effects and Mechanism of Mandarin (*Citrus reticulata* L.) Essential Oil against *Staphylococcus aureus*. Molecules.

[14] Perricone M., Arace E., Corbo M.R., Sinigaglia M., Bevilacqua A. (2015). Bioactivity of essential oils: A review on their interaction with food components. Front. Microbiol..

[15] Xia L., Li R., Tao T., Zhong R., Du H., Liao Z., Sun Z., Xu C. (2023). Therapeutic potential of *Litsea cubeba* essential oil in modulating inflammation and the gut microbiome. Front. Microbiol..

[16] Chen F., Wang Y., Wang K., Chen J., Jin K., Peng K., Chen X., Liu Z., Ouyang J., Wang Y. (2023). Effects of *Litsea cubeba* essential oil on growth performance, blood antioxidation, immune function, apparent digestibility of nutrients, and fecal microflora of pigs. Front. Pharmacol..

[17] Wang L., Hu W., Deng J., Liu X., Zhou J., Li X. (2019). Antibacterial activity of *Litsea cubeba* essential oil and its mechanism against Botrytis cinerea. Rsc Adv..

[18] Nguyen H.V., Caruso D., Lebrun M., Nguyen N.T., Trinh T.T., Meile J.C., Chu-Ky S., Sarter S. (2016). Antibacterial activity of *Litsea cubeba* (Lauraceae, May Chang) and its effects on the biological response of common carp *Cyprinus carpio* challenged with *Aeromonas hydrophila*. J. Appl. Microbiol..

[19] Bai M., Dai J., Hu W., Li C., Cui H., Lin L. (2023). The inhibition effect and mechanism of *Litsea cubeba* essential oil on the hemolytic activity of listeriolysin O. Food Biosci..

[20] Ho D.V., Hoang H.N.T., Nguyen N.H., Do H.B., Vo H.Q., Le A.T., Le T.Q., Pham T.V. (2023). GC-MS Characterization, in Vitro Antioxidant and Anti-Inflammatory Activities of Essential oil from the Leaves of *Litsea balansae* Lecomte. Nat. Prod. Commun..

[21] Si L., Chen Y., Han X., Zhan Z., Tian S., Cui Q., Wang Y. (2012). Chemical Composition of Essential Oils of *Litsea cubeba* Harvested from Its Distribution Areas in China. Molecules.

[22] Jia X., Li P., Wan J., He C. (2017). A review on phytochemical and pharmacological properties of *Litsea coreana*. Pharm. Biol..

[23] Gogoi R., Loying R., Sarma N., Munda S., Kumar Pandey S., Lal M. (2018). A comparative study on antioxidant, anti-inflammatory, genotoxicity, anti-microbial activities and chemical composition of fruit and leaf essential oils of *Litsea cubeba* Pers from North-east India. Ind. Crop Prod..

[24] Guo J., Li W., Wan S., Zhou J., Qin Z., Gao H. (2024). Antibacterial activity of *Amomum tsaoko* essential oil and its interaction with *Staphylococcus aureus*. LWT.

[25] Yang Y., Lin M., Feng S., Gu Q., Chen Y., Wang Y., Song D., Gao M. (2020). Chemical composition, antibacterial activity, and mechanism of action of essential oil from *Litsea cubeba* against foodborne bacteria. J. Food Process Pres..

[26] Shi C., Zhang X., Guo N. (2018). The antimicrobial activities and action-mechanism of tea tree oil against food-borne bacteria in fresh cucumber juice. Microb. Pathog..

[27] Liu S., Lan W., Xie J. (2024). Natural preservative *Litsea cubeba* essential oil: With emphasis on its biological activities, encapsulation methods and application in food preservation. Food Biosci..

[28] Rahman M.M., Wills R.B.H., Bowyer M.C., Golding J.B., Kirkman T., Pristijono P. (2020). Efficacy of Orange Essential Oil and Citral after Exposure to UV-C Irradiation to Inhibit *Penicillium digitatum* in Navel Oranges. Horticulturae.

[29] Mohamadi M., Mostafavi A., Shamspur T. (2011). Effect of Storage on Essential Oil Content and Composition of *Rosa damascena* Mill. Petals under Different Conditions. J. Essent. Oil Bear. Pl..

[30] Misharina T.A., Polshkov A.N., Ruchkina E.L., Medvedeva I.B. (2003). Changes in the composition of the essential oil of marjoram during storage. Appl. Biochem. Micro.

[31] Hąc-Wydro K., Flasiński M., Romańczuk K. (2017). Essential oils as food eco-preservatives: Model system studies on the effect of temperature on limonene antibacterial activity. Food Chem..

[32] Zheng L., Guo H., Zhu M., Xie L., Jin J., Korma S.A., Jin Q., Wang X., Cacciotti I. (2024). Intrinsic properties and extrinsic factors of food matrix system affecting the effectiveness of essential oils in foods: A comprehensive review. Crit. Rev. Food Sci..

[33] Badie F., Saffari M., Moniri R., Alani B., Atoof F., Khorshidi A., Shayestehpour M. (2021). The combined effect of stressful factors (temperature and pH) on the expression of biofilm, stress, and virulence genes in *Salmonella enterica* ser. Enteritidis and Typhimurium. Arch. Microbiol..

[34] Gutierrez A., Havelaar A.H., Schneider K.R. (2022). Antimicrobial Efficacy of Un-Ionized Ammonia (NH3) against Salmonella Typhimurium in Buffered Solutions with Variable pH, NH_3_ Concentrations, and Urease-Producing Bacteria. Microbiol. Spectr..

[35] Da Costa F.K.C., Cascaes J.M., Severo D.S., Santana M.B., Carciofi B.A.M., de Aragão G.M.F., Ienczak J.L. (2025). Influence of pH and oxygen levels on the antimicrobial efficacy of acetic acid against *Salmonella* Typhimurium. J. Appl. Microbiol..

[36] Tabanelli G., Montanari C., Patrignani F., Siroli L., Lanciotti R., Gardini F. (2014). Modeling with the Logistic Regression of the Growth/No Growth Interface of *Saccharomyces cerevisiae* in Relation to 2 Antimicrobial Terpenes (Citral and Linalool), pH, and a(w). J. Food Sci..

[37] Azadi A., Rafieian F., Sami M., Rezaei A. (2023). Fabrication, characterization and antimicrobial activity of chitosan/tragacanth gum/polyvinyl alcohol composite films incorporated with cinnamon essential oil nanoemulsion. Int. J. Biol. Macromol..

[38] Bajpai V.K., Sharma A., Baek K. (2013). Antibacterial mode of action of *Cudrania tricuspidata* fruit essential oil, affecting membrane permeability and surface characteristics of food-borne pathogens. Food Control..

[39] Yang H., Song L., Sun P., Su R., Wang S., Cheng S., Zhan X., Lü X., Xia X., Shi C. (2023). Synergistic bactericidal effect of ultrasound combined with citral nanoemulsion on Salmonella and its application in the preservation of purple kale. Ultrason. Sonochem..

[40] Lee W., Woo E., Lee D.G. (2016). Phytol has antibacterial property by inducing oxidative stress response in *Pseudomonas aeruginosa*. Free. Radic. Res..

[41] van Veldhuisen T.W., Altenburg W.J., Verwiel M.A.M., Lemmens L.J.M., Mason A.F., Merkx M., Brunsveld L., van Hest J.C.M. (2023). Enzymatic Regulation of Protein–Protein Interactions in Artificial Cells. Adv. Mater..

[42] Boni N., Shapiro L., Honig B., Wu Y., Rubinstein R. (2022). On the formation of ordered protein assemblies in cell–cell interfaces. Proc. Natl. Acad. Sci. USA.

[43] Nepal N., Arthur S., Haynes J., Palaniappan B., Sundaram U. (2021). Mechanism of Na-K-ATPase Inhibition by PGE2 in Intestinal Epithelial Cells. Cells.

[44] Vishnu N., Venkatesan M., Madaris T.R., Venkateswaran M.K., Stanley K., Ramachandran K., Chidambaram A., Madesh A.K., Yang W., Nair J. (2024). ERMA (TMEM_94_) is a P-type ATPase transporter for Mg^2+^ uptake in the endoplasmic reticulum. Mol. Cell.

[45] Rumbaugh K.P., Whiteley M. (2025). Towards improved biofilm models. Nat. Rev. Microbiol..

[46] Rodrigues Dos Santos E.A., Ereno Tadielo L., Arruda Schmiedt J., Silva Orisio P.H., de Cássia Lima Brugeff E., Sossai Possebon F., Olivia Pereira M., Gonçalves Pereira J., Dos Santos Bersot L. (2023). Inhibitory effects of piperine and black pepper essential oil on multispecies biofilm formation by *Listeria monocytogenes*, *Salmonella* Typhimurium, and *Pseudomonas aeruginosa*. LWT.

[47] Mu M., Oh J.K., Perez K., Zhou W., Wang X., Castillo A., Taylor M., Min Y., Cisneros-Zevallos L., Akbulut M. (2024). Effect of wax chain length on the adhesion dynamics and interfacial rigidity of *Salmonella* Typhimurium LT2. Surf. Interfaces.

[48] Liu M., Guo W., Feng M., Bai Y., Huang J., Cao Y. (2024). Antibacterial, anti-biofilm activity and underlying mechanism of garlic essential oil in water nanoemulsion against *Listeria monocytogenes*. LWT.

[49] Chen X., Zhang Y., Zu Y., Fu Y., Wang W. (2011). Composition and biological activities of the essential oil from *Schisandra chinensis* obtained by solvent-free microwave extraction. LWT—Food Sci. Technol..

[50] Shahar B., Chongtham N. (2024). Traditional uses and advances in recent research on wild aromatic plant Mentha longifolia and its pharmacological importance. Phytochem. Rev..

[51] Fathalizadeh M., Homayouni Tabrizi M., Tehranipour M. (2025). A novel alpha-terpineol-loaded niosome formulation coated with hyaluronic acid and evaluation of its anticancer properties in vitro. J. Mol. Liq..

[52] Cao C., Xie P., Zhou Y., Guo J. (2023). Characterization, Thermal Stability and Antimicrobial Evaluation of the Inclusion Complex of *Litsea cubeba* Essential Oil in Large-Ring Cyclodextrins (CD9–CD22). Foods.

[53] Elhabal S., Abdelaal N., Saeed Al-Zuhairy S., Elrefai M., Elsaid Hamdan A., Khalifa M., Hababeh S., Khasawneh M., Khamis G., Nelson J. (2024). Green Synthesis of Zinc Oxide Nanoparticles from Althaea officinalis Flower Extract Coated with Chitosan for Potential Healing Effects on Diabetic Wounds by Inhibiting TNF-α and IL-6/IL-1β Signaling Pathways. Int. J. Nanomed..

[54] Liu T., Kang J., Liu L. (2021). Thymol as a critical component of *Thymus vulgaris* L. essential oil combats *Pseudomonas aeruginosa* by intercalating DNA and inactivating biofilm. LWT.

[55] Taggar R., Jangra M., Dwivedi A., Bansal K., Patil P.B., Bhattacharyya M.S., Nandanwar H., Sahoo D.K. (2021). Bacteriocin isolated from the natural inhabitant of *Allium cepa* against Staphylococcus aureus. World J. Microbiol. Biotechnol..

[56] Ghaderi L., Aliahmadi A., Ebrahimi S.N., Rafati H. (2021). Effective Inhibition and eradication of *Pseudomonas aeruginosa* biofilms by *Satureja khuzistanica* essential oil nanoemulsion. J. Drug Deliv. Sci. Tec..

[57] Tang W., Zhang Z., Nie D., Liu S., Li Y., Liu M., Zhang Y., Ou N., Li Y. (2023). Selective antibacterial activity of C*itrus Medica limonum* essential oil against *Escherichia coli* K99 and *Lactobacillus acidophilus* and its antibacterial mechanism. LWT.

